# How Does a Delicate Insect Wing Resist Damage? Chitin Orientation Is Adapted to the Mechanical Demands at the Nanoscale

**DOI:** 10.1002/adma.202503941

**Published:** 2025-08-01

**Authors:** Chuchu Li, Jiliang Liu, Manfred Burghammer, Chao Wan, Stanislav N. Gorb, Clemens F. Schaber

**Affiliations:** ^1^ Functional Morphology and Biomechanics Zoological Institute Kiel University 24098 Kiel Germany; ^2^ European Synchrotron Radiation Facility (ESRF) CS 40220 Grenoble Cedex 9 38043 France; ^3^ Department of Mechanics School of Aerospace Engineering Beijing Institute of Technology Beijing 100083 China

**Keywords:** chitin fiber orientation, finite element analysis, framed shear structure, locust wing, X‐ray scattering

## Abstract

Insect wings achieve an extraordinary balance between structural robustness and lightweight flexibility, enabling efficient and durable flight. This performance arises from their hierarchical composite architecture, where nanoscale chitin fiber orientations play a critical role in adapting to complex mechanical demands. Using scanning X‐ray micro‐ and nano‐diffraction, the spatial distribution and orientation of chitin fibers in the hindwing of the desert locust *Schistocerca gregaria* are systematically mapped. These findings reveal two distinct and functionally adaptive chitin orientation patterns in the membranes that vary regionally, optimizing mechanical resilience and deformation control. Finite element simulations further demonstrate how these nanoscale structural adaptations enhance crack resistance, structural integrity, and elastic strain energy distribution, reinforcing vein‐membrane connections for sustained functionality under various loadings. By integrating high‐resolution structural analysis with computational modeling, this study uncovers the sophisticated biomechanical strategies that enable insect wings to endure extreme flight conditions. These insights provide a foundation for bioinspired designs in micro air vehicles and advanced fiber‐reinforced materials.

## Graphical abstract

Nanoscale chitin fiber orientations in the insect wing play a critical role in adapting to complex mechanical demands. These findings reveal two distinct and functionally adaptive chitin orientation patterns in the membranes that vary regionally, optimizing mechanical resilience and deformation control. These insights provide a foundation for bioinspired designs in micro air vehicles and advanced fiber‐reinforced materials.

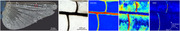

## 1 Introduction

Insect wings appear as delicate and fragile structures, yet they efficiently withstand internal and external mechanical stress and generate aerodynamic forces substantially larger than the animals’ weight for millions of loading cycles during flight.^[^ ^1^, ^2^, ^3^ ^]^ The wings are passive structures actuated by muscles located inside the insect´s thorax. The wings mainly consist of cuticle, a composite material of proteins and embedded reinforcing fibers of the polysaccharide chitin.^[^ ^4^ ^]^ The thin laminar wing membrane is strengthened by a framework of tube‐shaped hollow longitudinal and connecting cross veins. In living insects, the longitudinal veins are filled with the hemolymph fluid that supplies the living tissue with nutrients. The veins divide the membrane into polygonal cells.

In flight, insect wings can experience different types of dynamic loading and actively or passively change their shape.^[^ ^5^, ^6^ ^]^ Such deformation is determined partly by forces driven by muscles and connected skeletal components at the wing base, and partly by external forces, such as aerodynamics and collisions.^[^ ^7^ ^]^ The morphology and mechanical properties of the wings are suggested to adapt to external forces and help to achieve passive deformation. This level of automatic shape control in propulsive appendages is remarkable,^[^ ^8^ ^]^ making insects a popular model for the inspiration of micro air vehicles (MAVs).^[^ ^9^ ^]^

Many studies have focused on the functional morphology, structure, mechanical properties, dynamic kinematics, and simulation related to the remarkable flight performance achieved by insect wings.^[^ ^7^, ^10^, ^11^, ^12^, ^13^, ^14^, ^15^, ^16^, ^17^, ^18^, ^19^, ^20^, ^21^, ^22^, ^23^ ^]^ However, little is known about the orientation of fibers in insect wing materials and their mechanical consequences. Hypotheses suggest that a preferred chitin fiber orientation impacts the mechanical properties of the wing membrane.^[^ ^24^ ^]^ Yet, studies using polarization microscopy found no evidence of preferred fiber orientation in locust wings.^[^ ^7^ ^]^ Directionally oriented fibrous components were detected in the wing membranes of a cicada and a butterfly species using scanning electron microscopy,^[^ ^25^, ^26^ ^]^ but these results do not reveal the exact fiber orientation.

Two factors may explain this knowledge gap. First, researchers for a long time have believed that the membrane of insect wings is made of epicuticle only, which consists of proteins and wax without chitin.^[^ ^7^ ^]^ Later, it has been reported that the wing membrane of the dragonfly *Libellula basilinea* ^[^ ^27^ ^]^ and the house cricket *Acheta domesticus* ^[^ ^28^ ^]^ contain procuticle, which is made of exo‐ and endocuticle and well accepted to contain chitin.^[^ ^29^ ^]^ Only recently, using scanning pyroelectric microscopy (SPEM), the presence of chitin in the wing membrane of houseflies and wasps has been confirmed.^[^ ^30^ ^]^ Second, the contribution of fiber orientation to mechanical behavior in insect cuticle might have been overlooked. In materials science, many studies have focused on artificially made fiber‐reinforced composites with different fiber type, size, and density.^[^ ^31^ ^]^ In addition, related efforts in biomimetic materials (e.g., based on bagworm silk) have shown how fiber orientation and hierarchical architecture can improve toughness and strength.^[^ ^32^, ^33^ ^]^ For insect cuticle, however, only one classical study illustrated the directional properties of Young's modulus and shear modulus as a function of fiber orientation patterns,^[^ ^4^ ^]^ and only two recent studies showed strong contributions of the orientation of the nanofibers in locust leg cuticle for different mechanical loads.^[^ ^34^, ^35^ ^]^ Therefore, the presence of chitin and a possible specific chitin fiber orientation has to be considered a crucial factor for the high stability and durability of insect wings during flight, by specifically controlling the wing material properties.

In this study, we have employed scanning X‐ray micro‐ and nano‐diffraction, which covers the wide‐angle scattering regime, to detect the chitin fiber orientation and distribution in the hindwing of the desert locust *Schistocerca gregaria*, a well‐established model species for insect wing studies.^[^ ^10^, ^13^, ^20^, ^22^, ^23^ ^]^ Additionally, we conducted finite element analysis to explore how the observed chitin fiber orientations influence mechanical performance. These simulations revealed the interplay between fiber alignment and critical factors such as crack resistance, deformation, and elastic strain energy, providing a comprehensive understanding of how structural adaptations optimize wing function during flight. Our results provide incredibly detailed insights into chitin fiber orientation in the hindwing membrane of desert locusts, revealing how the orientations reinforce vein‐membrane connections and the membrane itself for specific demands of mechanical stability and flexibility of the wing during flight.

## 2 Results

### 2.1 X‐Ray Diffraction Patterns of Chitin

**Figure 1:**
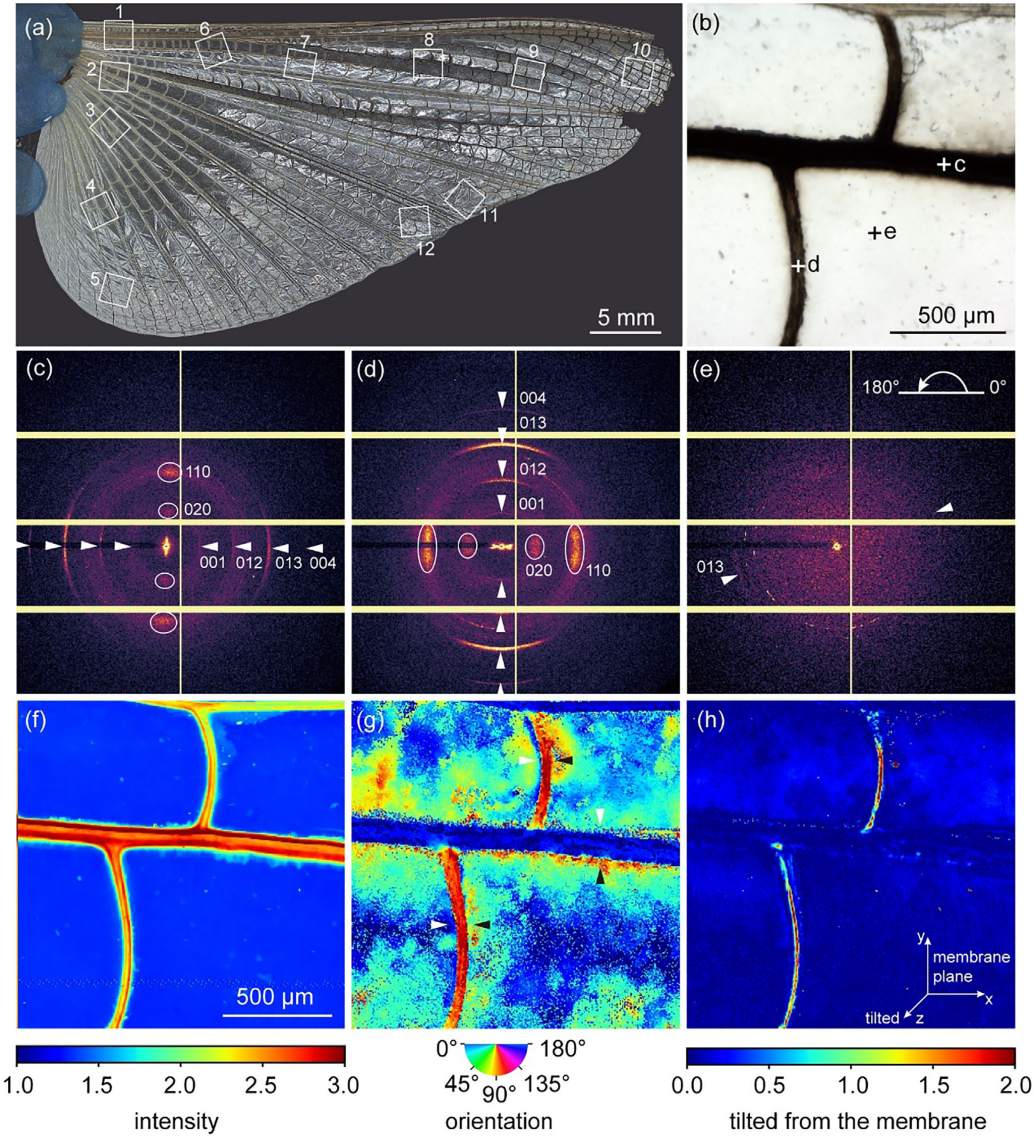
Locust hindwing specimens and averaged X‐ray diffraction patterns from different regions. a) Unfolded right hindwing of a female locust. The 12 different regions were investigated. b) Transmitted light micrograph of the piece of the hindwing at region 8. c–e) Averaged X‐ray microdiffraction pattern of the longitudinal vein (c), cross vein (d), and membrane (e). The sharper WAXS signals corresponding to the equatorial 020 and 110, and the meridional 001, 012, 013, and 004 reflections of fibrous α‐chitin are highlighted by white arrowheads. Note that the longitudinal orientation of the crystalline chitin chains is perpendicular to the equator of the WAXS signals (020 and 110), and parallel to the α‐chitin signals (001, 012, 013, and 004). White arrowheads point to the reflections off the equator as a guide for the eye, showing the fiber orientation. f–h) Intensity and orientation of chitin signal of region 8 from scanning micro‐focus X‐ray diffraction (image resolution ≈2 µm). Intensity map (f) showing the distribution of chitin based on wide‐angle X‐diffraction. g) Orientation map indicating the directionality of crystalline chitin chains. The arrowheads point to the angular 90° switch of fiber angle between the two sides of the veins. h) Intensity map showing the orientation of crystalline chitin chains based on the level that it is tilted from the membrane plane.

The WAXS patterns from 12 different regions of the hindwing specimens (**Figure** **1**a), including the longitudinal vein, cross vein, and membrane, specifically show the typical fiber diffraction pattern of chitin, which is confirmed by several characteristic diffraction peaks. The major reflections observed include 020 (≈5.6 nm^−1^), 110 (≈13.8 nm^−1^), 001 (≈6.4 nm^−1^), 012 (≈13.2 nm^−1^), 013 (≈18.6 nm^−1^), and 004 (≈24.3 nm^−1^), which align well with the reported Q‐values in previous crystallographic studies of α‐chitin.^[^ ^36^, ^37^, ^38^ ^]^ Among them, the 013 reflection was selected for orientation analysis due to its favorable signal‐to‐noise ratio and sensitivity to chitin alignment along the fiber axis. This reflection was clearly detected in both the longitudinal veins and the membranous regions. Its intensity was consistently higher and sharper in the veins, which we attributed to the greater cuticle thickness and higher order of crystallinity. In contrast, the membrane exhibited a broader and weaker 013 peak, consistent with a lower overall crystallinity. Nonetheless, the directional distribution of this signal in the membrane remained consistent and anisotropic, supporting the presence of organized chitin orientation even in these thin and compliant regions.

### 2.2 Chitin Fiber Distribution and Orientation Resolved Using Microfocus Mapping

The detected chitin signal intensity was highest close to the edges of the longitudinal veins. In the center of the longitudinal veins, the intensity was lower. This distribution points to the hollow tube shape of the longitudinal veins, which is visible microscopically in cross‐section preparations and similar to the data previously found for the hollow shaft of adhesive spider setae.^[^ ^36^ ^]^ In the cross veins, the intensity of the 013 signal was weaker, depicting their lower mass fraction or lower degree of crystalline order. Furthermore, lower chitin‐caused diffraction intensities were found at the transition zones between veins and membranes, while the lowest one was found in the membrane. The variation in diffraction intensity observed across different structural domains of the wing appears to correlate with local cuticle thickness. For instance, the longitudinal veins, which typically have diameters of 100–150 µm,^[^ ^10^ ^]^ exhibited the strongest diffraction signals. The intermediate zone between vein and membrane, with an average thickness of ≈50 µm, showed reduced intensity. In contrast, the membrane itself, which is significantly thinner (≈1.5–4 µm,^[^ ^7^ ^]^ produced the weakest signal. These observations suggest that reduced sample thickness leads to lower diffracted signal intensity, likely due to a smaller illuminated volume and reduced crystalline material along the beam path. Dispersed spots with higher intensity within the membrane areas indicate setae standing off vertically from the wing surface in parallel with the incoming focused X‐ray beam (Figure 1f).

The preferred directional orientation of the chitin fibers within the membrane plane is shown in Figure 1g. The chitin fibers in the veins were convergently oriented along the long axes of the veins. The directionality of the fibers in the membrane varied; however, it seems to follow a specific pattern. For example, in region 8, the chitin fibers in the middle region of each membrane cell had the same orientation as the longitudinal veins (Figure 1g). Approaching the longitudinal vein from below, the chitin main orientation gradually changed from parallel to approximately orthogonal to the longitudinal vein, while approaching the longitudinal vein from up, the chitin orientation mainly remains parallel to the longitudinal vein. Additionally, the chitin fiber orientation in the vicinity of the cross veins differs by ≈90° between the two sides of the veins, e.g., with angles of ≈170° to the cross veins on the proximal side and 80° on the distal side of the cell (Figure 1g, white and black arrows). Interestingly, such a switch of angle was not found toward the upper longitudinal vein. Figure 1h presents the intensity map showing the orientation of crystalline chitin chains based on the level at which it is tilted from the membrane plane. The chitin fibers of the longitudinal vein and membrane were arranged within the membrane plane, while those of the cross veins were partly tilted out of the membrane. The tilted‐out signals of cross veins very likely resulted from the periodicity of the hump‐pattern morphology that was only found in the cross veins.^[^ ^23^ ^]^

### 2.3 Chitin Fiber Orientation Resolved Using Nanofocus Mapping

**Figure 2:**
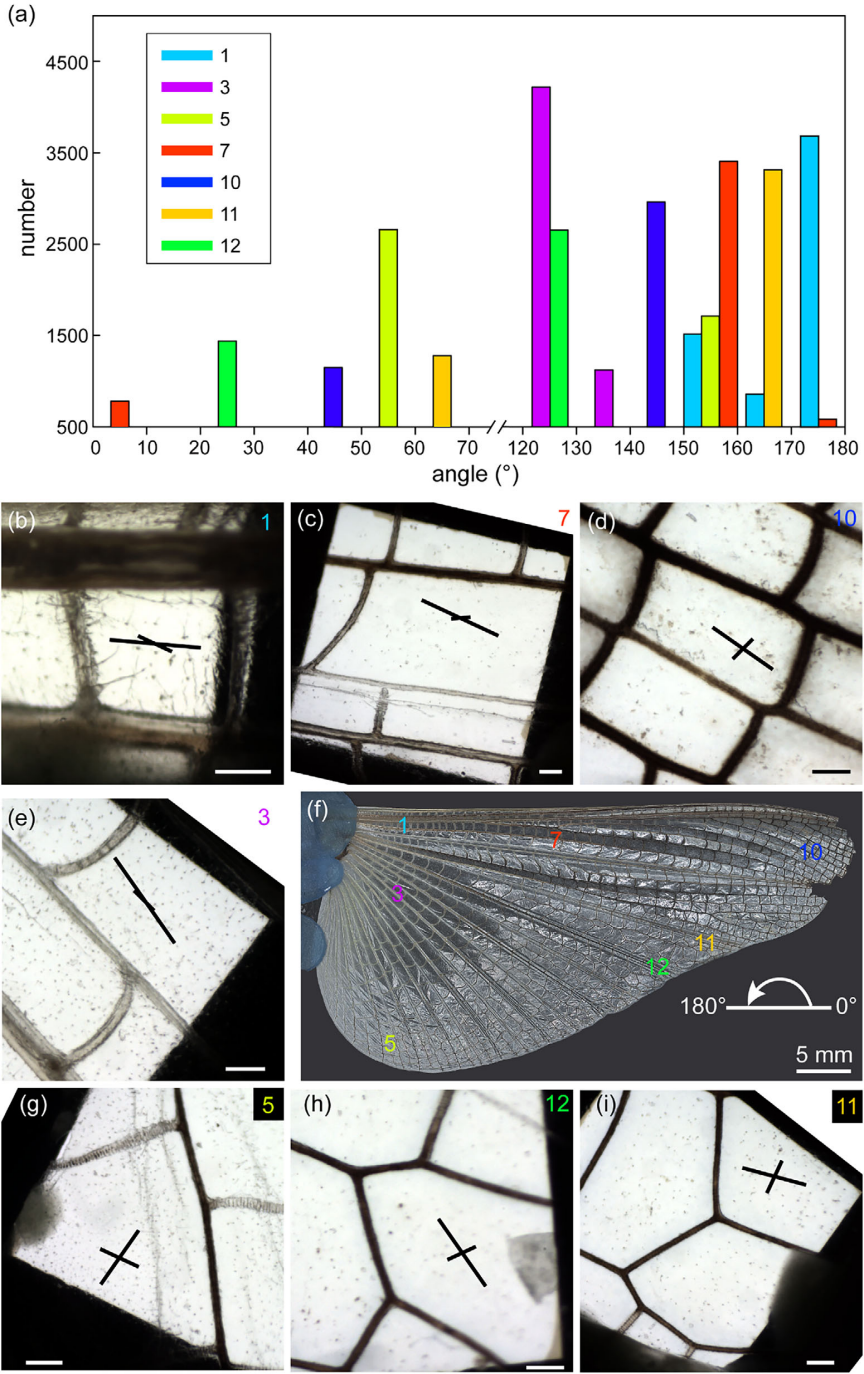
Preferred chitin fiber orientations in different regions of the locust hindwing membrane. The labels of the photomicrographs correspond to the sample numbers in Figure 1a. The black lines in the membrane cells indicate the preferred directions of chitin fiber orientation in each specimen. The length of the lines corresponds to the number count in the diagram. For region 1 b) and region 7 c), three lines corresponding to the preferred orientations are shown, although the individual lines may overlap due to similar orientations. The shortest line lies between the two longer ones, and no zoom‐in is included to preserve the overall visual pattern. Scale bars: b–e, g–i, 200 µm.

Using WAXS of nanodiffraction, we have successfully measured the chitin‐oriented angles in 7 different areas, which are situated at the base, middle, and tip regions of the hindwing along both spanwise and chordwise directions, namely regions 1, 3, 5, 7, 10, 11, and 12 (**Figure** **2**f). For each region, we have scanned 6400 points in an area of 400 µm^2^, while the distance between every two points is 250 nm. We subsequently analyzed the fiber‐oriented angles of all the scans and counted the numbers by every 10 degrees for each region. To avoid the effects of dust and noise, we presented the angles in Figure 2a only when they were >500 numbers for each region. All of the presented angles were normalized based on the coordinate system shown for the whole hindwing (Figure 2f). Scale black lines were drawn on each membrane specimen, showing the angles and scaled by the numbers of each angle of that region. Our results clearly show that the level of anisotropic chitin orientation on the wing membrane differs between areas. At the base (region 1) and middle areas (region 3 and 7), the majority of the chitin fibers are arranged along the radial direction, namely along their neighboring longitudinal veins. The variation of chitin‐arranged angles was <30°. However, at the tip areas (regions 5, 10, 11, and 12), there were two peaks of the fiber‐oriented angles of each region, while one peak angle was nearly perpendicular to the other. Based on overall wing coordinates, a clockwise rotation pattern of chitin preferred angle has been found along both spanwise (Figure 2b–d) and chordwise directions (Figure 2b,e,g). When considering the chitin orientation within each membrane cell, no specific pattern was found.

### 2.4 Numerical Modeling of Mechanical Behaviors of Different Membrane Patterns

**Figure 3:**
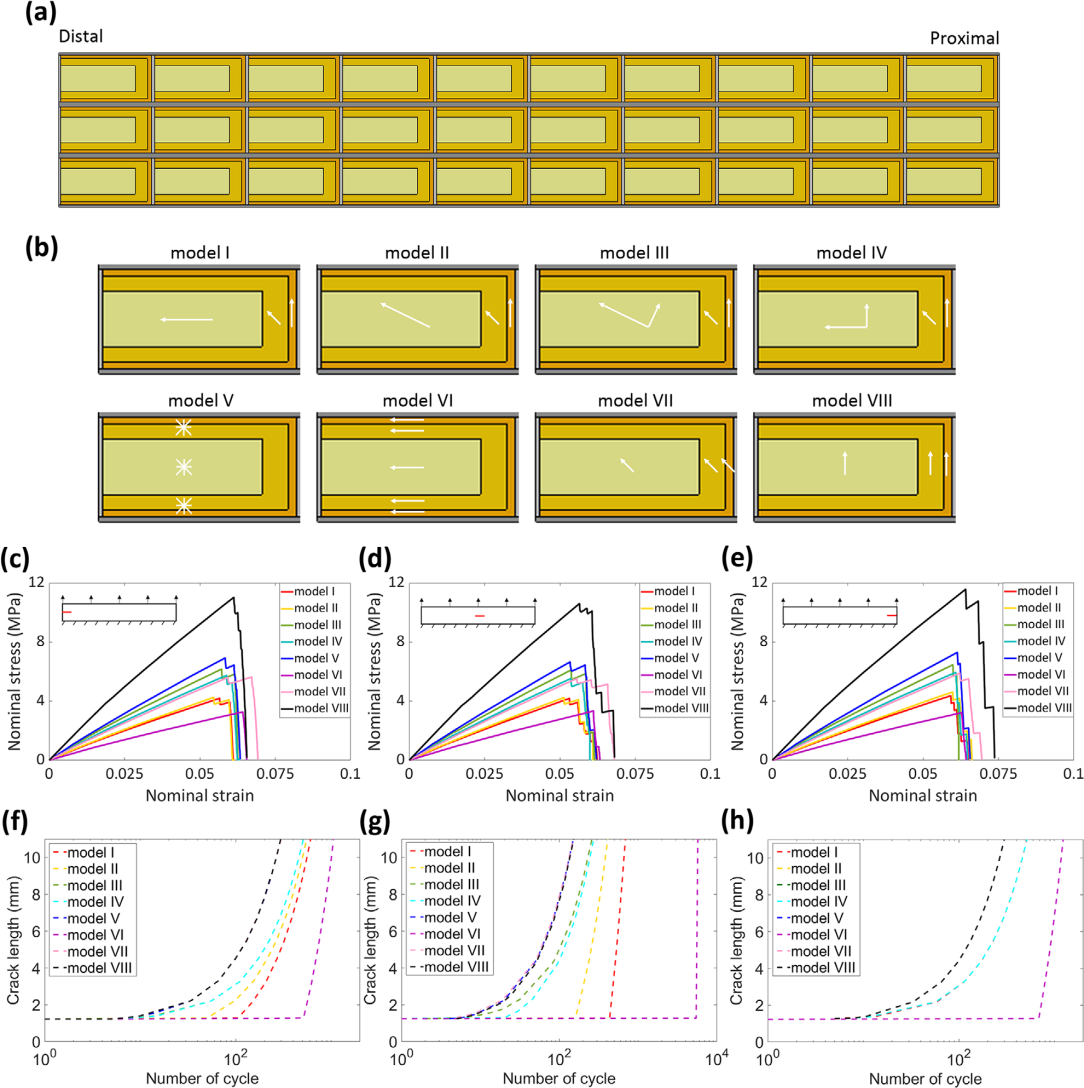
Simulations of the eight different membrane patterns with a crack propagating from different locations. a) The whole 2D model of the hind wings with regularly closed cells. The dark and light gray colors represent the longitudinal and cross veins, respectively. The membrane includes outer (orange), middle (dark yellow), and inner portions (light yellow), which have different fiber alignments. b) Diagram of the eight membrane models with different fiber alignments in the three membrane portions. The fiber directions of the membrane are marked by white arrows, while the asterisk in model V means that the membrane contains randomly oriented fibers. c–e) The strain‐stress curves of the eight membrane models (I–VIII) during the crack propagation under quasi‐static loads from the distal side (c), middle side (d), and proximal side (e). Schematic diagrams are displayed at the top‐left corner of each subgraph, where red lines represent crack locations. f–h) The crack growth under cyclic tension loading of the eight membrane models (I–VIII) with the initial crack propagating from the distal side (f), middle side (g), and proximal side (h). Some curves overlap because of similar results like models IV, VII, and VIII in (f–h), models I–IV in (h).

Based on the experimental results, we have created eight numerical models of membrane patterns to analyze their mechanical performances, including the resistance ability of crack propagation, failure possibility, deformation, and elastic strain energy. Four natural membrane patterns (**Figure** **3**b, models I–IV) and four artificial membrane patterns (Figure 3b, models V–VIII) were quantitatively compared. The four natural membrane patterns in the simulation models I, II, III, and IV refer to the membranes observed at locations 1 and 3, at location 7, at location 5, and at locations 10, 11, and 12, respectively (shown in Figure 2). The stress‐strain curves of each model during the crack propagation of quasi‐static and cyclic tension loadings are shown in Figure 3c–e and f–h, respectively. It was indicated that the highest stresses of each model during the crack propagation under the quasi‐static tension were similar regardless of initial crack location (difference was <9%). Moreover, similar results were achieved from the simulations of the three crack locations, in that the membranes in model VIII all suffered higher stress (>10 MPa) than those in other models (3–7 MPa). The best resistance performance against crack propagation under quasi‐static tension resulted from the membrane with all fibers along the cross veins (model VIII) and followed by the isotropic membrane with randomly oriented fibers (model V). Furthermore, the four natural membrane patterns (models I–IV) showed different crack resistance abilities: the membranes near the outer margin (models III and IV) can suffer 30–50% more stress than those near the wing base (models I and II) during crack propagation under quasi‐static tension. The membrane with 45‐degree tilted fibers (model VII) also revealed similar resistance properties as the natural membrane near the outer margin (models III and IV). The processes of the crack propagation in all eight FE models under the quasi‐static tension were shown in Videos S1–S3 (Supporting Information). On the contrary, the four natural membrane patterns (models I–IV) under cyclic tension loads (shown in Figure 3f–h) revealed a significantly better crack resistance than the three artificial patterns (models V, VII, and VIII). The artificial membrane pattern with all fibers perpendicular to the cross veins (model VI) exhibited the best crack resistance ability under cyclic load, especially the cycle number before the onset of crack growth.

**Figure 4:**
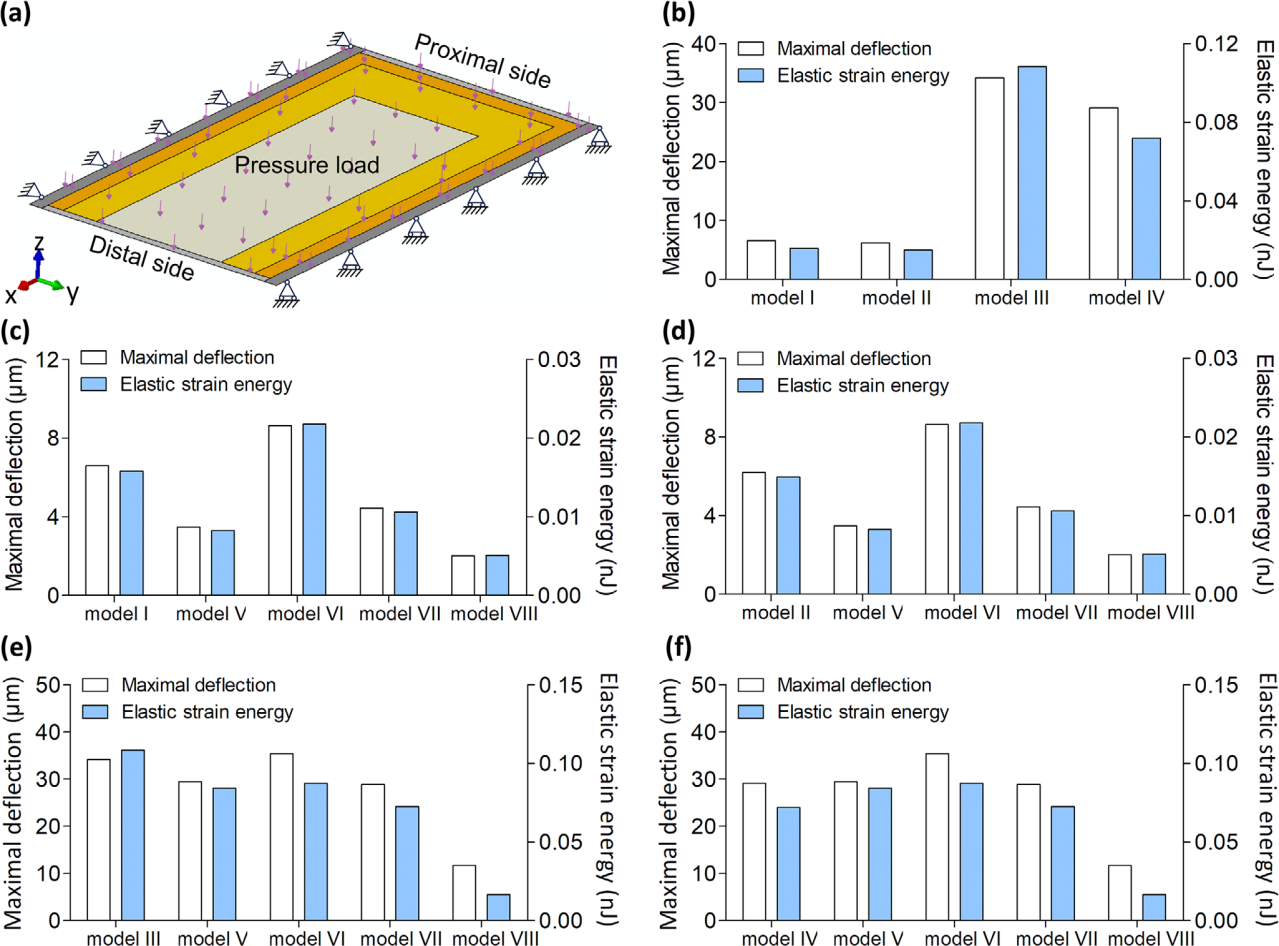
Simulation of the one‐cell membrane models under 12 Pa pressure load. a) Diagram of the model and its boundary conditions. b–f) Comparisons of the maximal deflection and elastic strain energy of the eight one‐cell membrane models: (b) between the four natural models with natural fiber orientations (models I–IV), (c) between model I and the four artificial models (models V–VIII), (d) between model II and the four artificial models (models V–VIII), (e) between model III and the four artificial models (models V–VIII), and (f) between model IV and the four artificial models (models V–VIII).

The mechanical functions of the membrane under the pressure load of airflow were further compared among the eight one‐cell models (**Figure** **4**), including both the bending deflection and elastic strain energy under the pressure load of 12 Pa and the failure factors under the 120 Pa pressure load. First, both the bending deflection and elastic strain energy results of the cells near the wing base (i.e., models I and II) were much lower than those of the cells near the margin (i.e., models III and IV) under the 12 Pa pressure load, shown in Figure 4b. Compared to models I and II, all the artificial models with the same membrane thickness demonstrated considerably lower bending deflection and elastic strain energy results, except for model VI (Figure 4c,d). On the contrary, three artificial models (V, VI, and VII) with the same membrane thickness showed similar bending deflection and elastic strain energy as those of models III and IV, while model VIII showed obviously lower results (Figure 4e,f).

**Figure 5:**
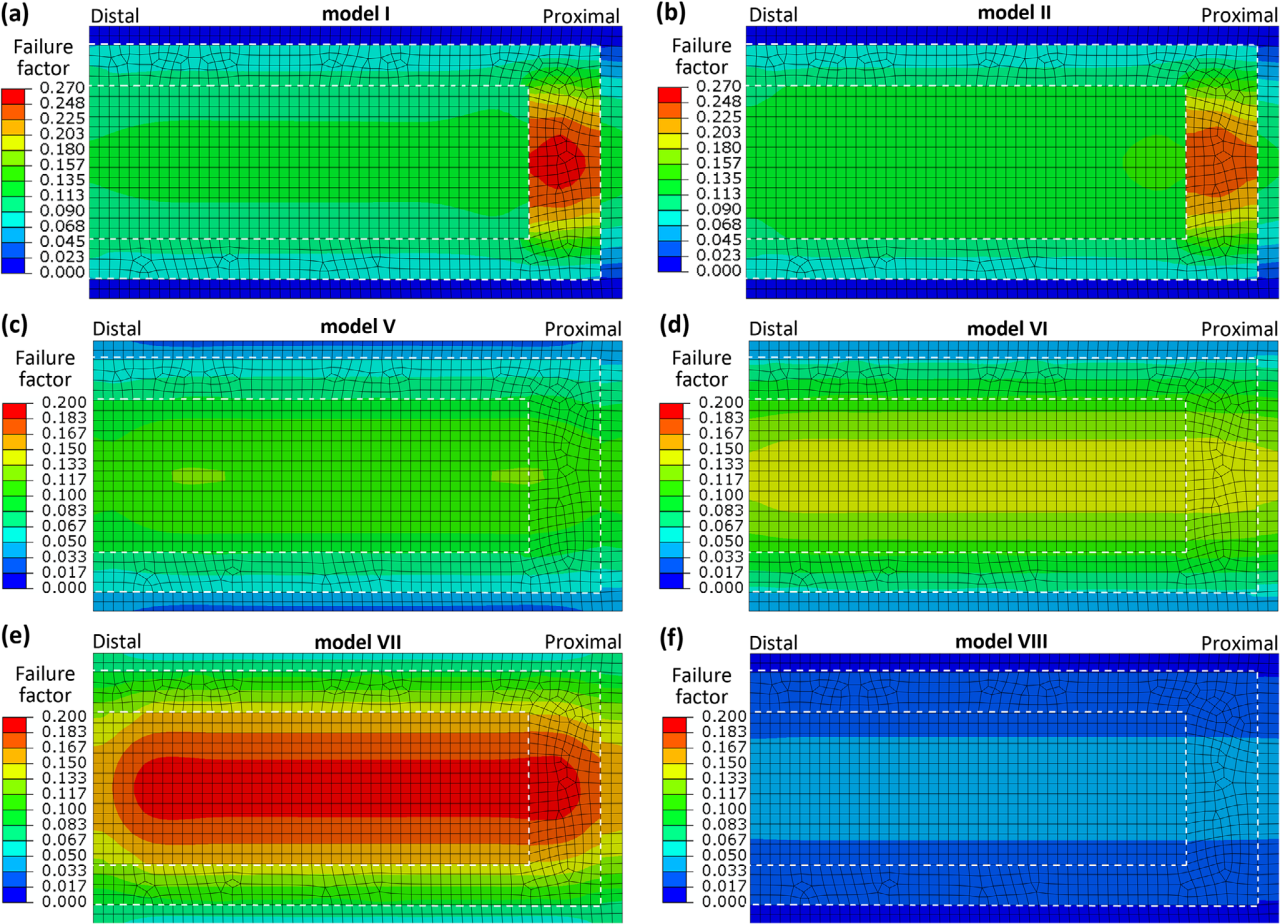
The failure factor results of the four artificial and two natural one‐cell membrane models with the same thickness of 4 µm under 120 Pa pressure load. The boundary lines between the three membrane portions are marked by white dashed lines.

**Figure 6:**
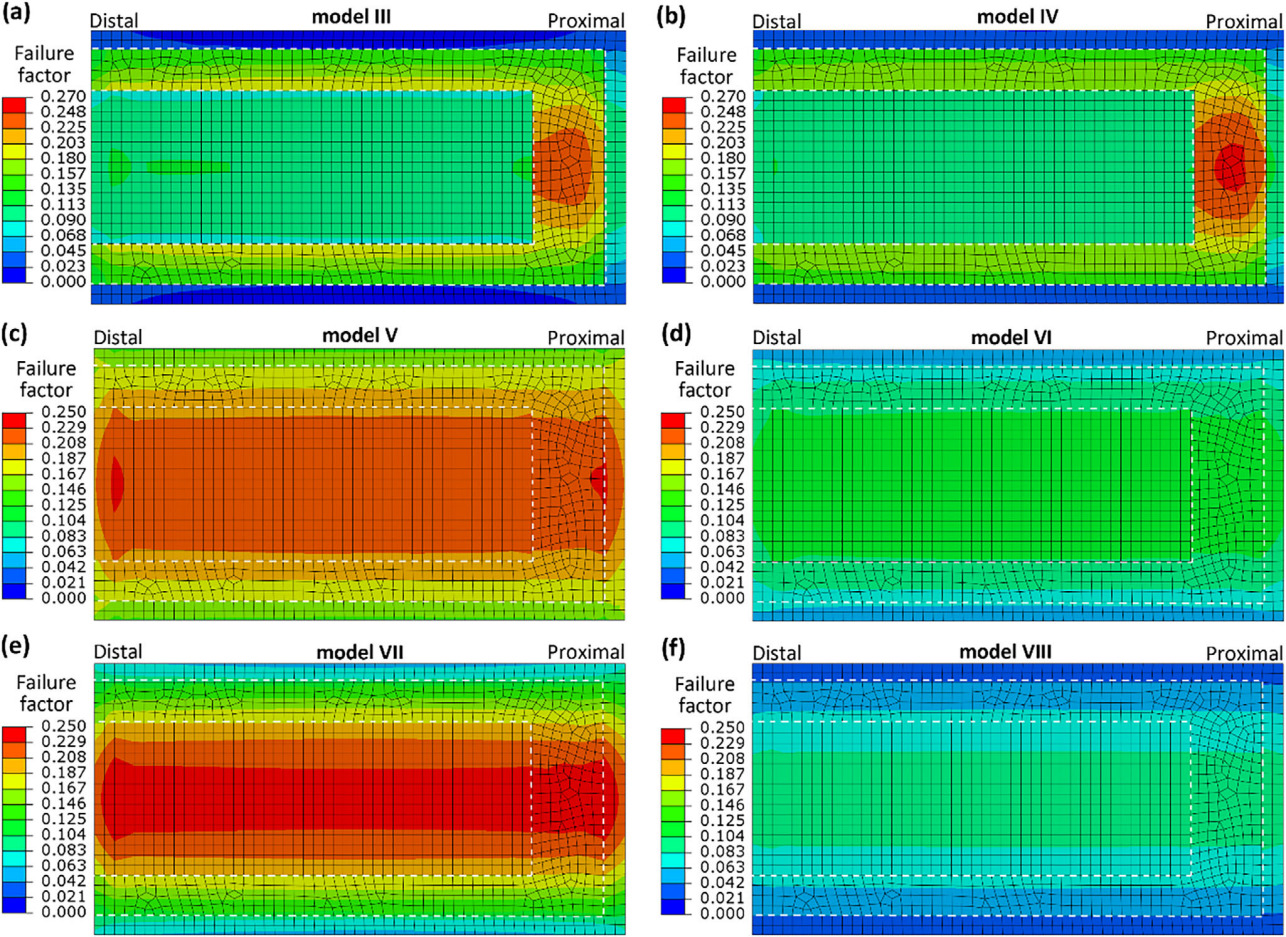
The failure factor results of the four artificial and two natural one‐cell membrane models with the same thickness of 1.5 µm under 120 Pa pressure load. The boundary lines between the three membrane portions are marked by dashed lines.

Moreover, the distribution of the failure factor in the membrane under the higher‐pressure load (120 Pa) was compared among the natural and artificial cell models with the same membrane thickness (**Figures** **5** and **6**). For the comparisons of the four artificial models and two natural models (I and II), high failure factors (>0.19) occurred in both the inner and middle portions of the membrane of model VII, while some higher failure factor (0.25–0.26) was found in the middle portions of the membrane in models I and II. For the comparisons of the four artificial models and the other two natural models (III and IV), both models V and VII demonstrated a large region of high failure factor (> 0.24) while models III and IV had small local regions of high failure factor in the middle portions of the membrane.

## 3 Discussion

Insect wings are supported by some longitudinal veins, often connected by short cross veins, forming a kind of wing skeleton. Our results demonstrate that chitin fibers in longitudinal and cross veins are oriented along their respective lengths. This is very likely a result of the adaptation to the mechanical functions of wing veins, enabling the hindwing of locusts to unfold along both chordwise and spanwise directions. To fulfill the function, the veins should be able to withstand buckling and bending failures, which rather often happen to long, rod‐shaped structures. Cuticle is an anisotropic biological material with the highest elastic modulus in the direction along the fiber length.^[^ ^4^, ^34^, ^35^ ^]^ Therefore, chitin fibers oriented along the length of the veins would result in a higher elastic modulus in the vein length direction and reduce the risk of buckling and bending damage to the wings. Furthermore, our results give insight into the spatial orientation of the fibers instead of 2D orientation within the wing membrane plane. For example, chitin fibers of the longitudinal vein are oriented parallel to the membrane plane, while those of the cross veins are spatially oriented along the length of the cross vein, combined with tilted orientation out of the membrane plane. However, the cuticle is a laminated composite material with all the fibers embedded parallel to the sublayers. It is unlikely that the chitin fibers can go through the sublayers. Therefore, the spatial chitin fiber orientation of the cross vein is very likely to result from the morphology of the cross veins. A recent study has shown that the cross veins of locust hindwings have humps along their length.^[^ ^23^ ^]^ These humps stand out from the cross vein and thus could bring the embedded chitin fibers tilted out of the membrane without tilting from the laminations.

The wing membrane can function not only as an aerodynamic surface but also as a mechanical support against collisions.^[^ ^24^, ^39^ ^]^ It has also been suggested that if the chitin in the membrane has preferred aligned angles, these angles would be running parallel to the frame's diagonals,^[^ ^24^ ^]^ because the forces passed to the membrane would run parallel to the frame's diagonals according to Wanger Tension theory.^[^ ^40^ ^]^ However, this study shows that reality is more complex. The chitin orientation is based on the overall wing coordinate instead of a single cell. Since the level of anisotropic chitin orientation is associated with regions with different thicknesses of veins, one can assume that the contributing ratio of membrane and vein differs between regions, and therefore, at the outer margin with smaller veins, the chitin fiber orientation in the membrane should adapt to serve as an additional support for the veins.

In addition to fiber orientation, the degree of crystallinity may also contribute to local mechanical behavior. The sharper and more intense diffraction peaks observed in the veins suggest a higher degree of crystallinity compared to the membranes, potentially reinforcing their structural stiffness. Although our analysis focused on fiber orientation, spatial variation in crystalline order may provide an additional mechanism for mechanical tuning in insect wings. This possibility merits further investigation in future studies.

The simulation results additionally show that the natural patterns of the membrane have offered the hindwing multiple mechanical functions, which seem to have evolved as a compromise between sufficient resistance against crack/failure and functional elastic deformation for flying. Regardless of crack location, the natural membrane patterns (models I–IV) have stronger resistance abilities against crack propagation under the quasi‐static loads than the membrane with all fibers aligned along the longitudinal veins (model VI), and weaker crack resistance than the membrane model VIII with fibers aligned along the cross veins (Figure 3c–e). The other two artificial membrane models (V and VII) provide similar anti‐crack propagation ability under the quasi‐static loads as the natural membrane patterns. Some sensitivity analyses (shown in Figure S1, Supporting Information) further demonstrated that this crack resistance performance does not vary significantly with the change of boundary lines between the three membrane portions, which means that this resistance ability is stable and does not disappear with the change of the membrane portion dimension. By contrast, the four natural membrane patterns can offer better fatigue resistance properties than the artificial membrane patterns (models V, VII, and VIII) with both delayed onset of crack growth and higher cycle number before failure. The best crack resistance under the cyclic loads is also found in the artificial membrane pattern with all fibers aligned along the longitudinal veins (model VI). The different fatigue behaviors are suggested to be caused by the inclusion of longitudinally aligned fibers. According to fatigue mechanics,^[^ ^41^, ^42^ ^]^ both the onset and growth rate of the crack during fatigue loadings are positively related to the strain energy release rate range, which is lower in the longitudinally aligned fibrous portion because of its anisotropic properties and lower stress range. Although the artificial models V, VII, and VIII offer similar or stronger crack resistance against the quasi‐static loads as the four natural membrane patterns (models I–IV), they do not have good fatigue resistance.

In addition, the eight membrane patterns under the physiological loads of the flapping flight were compared, and revealed that the four artificial membrane patterns have different mechanical properties. Compared to the natural membrane patterns, the membrane patterns (model VIII) have smaller deformation and lower elastic strain energy. On the contrary, the other two artificial membrane models (V and VII) cannot satisfy both the requirements of enough deformation and low failure. Specifically, model V demonstrates lower failure possibility and insufficient deformation compared to models I and II (i.e., membrane patterns near the wing base). Compared to models III and IV (i.e., membrane patterns near the outer margin), model VII shows enough deformation and high failure possibility. Some sensitivity analyses of the three membrane portion dimensions have also been performed, revealing that a small change in the portion dimension does not greatly affect the bending performance (shown in Figure S2, Supporting Information). In brief, artificial membrane patterns cannot offer acceptable multi‐functions of crack resistance, sufficient deformation, and low failure like natural membrane patterns. It was demonstrated that wing flexibility plays a significant role in insect flight efficiency. For example, a compliant wing can reduce energy expenditure by almost 25% compared to a rigid wing by storing elastic strain energy in the negative work period of a single wingbeat and subsequently releasing it in its positive work period.^[^ ^43^, ^44^ ^]^ A similar mechanism of energy efficiency improvements has also been found in the flapping flights of the fruit fly *Drosophila* and hover fly.^[^ ^5^, ^45^ ^]^ Furthermore, different deformation abilities of the four natural patterns (i.e., stiffer near the wing base and softer near the outer margin) can help the hind wing to form the “umbrella effect” (camber), which was suggested to be the key role for the downstroke of locust flapping flight.^[^ ^20^, ^46^ ^]^ In addition, the deformability of the natural membranes can contribute to the gliding flight performance of the locust by holding enough vortex on the corrugated wing.^[^ ^47^ ^]^ From the perspective of wing flexibility, either the elastic deformation or failure factor level of the four artificial models cannot be accepted, although they exhibit similar or stronger crack resistance (model VI against the cyclic loads and models V, VII, and VIII against the quasi‐static loads).

The region‐specific fiber alignment observed in locust wings provides valuable design principles for synthetic fiber‐reinforced composites. By gradually varying orientation and introducing local anisotropy, biological structures can combine flexibility, damage resistance, and mechanical efficiency, particularly in thin and deformable systems. Although replication of such precise orientation control in artificial materials remains challenging, recent studies have shown promising results using architected fiber networks and hierarchical interfaces. For example, Chen et al. designed a gradient composite that mimics biological energy absorption and achieved improved toughness.^[^ ^48^ ^]^ Xiao et al. further demonstrated multiscale toughening through local fiber orientation control.^[^ ^49^ ^]^ Our study adds a structural‐level understanding of natural fiber orientation at micrometer to nanometer scales of insect wing membrane and may help guide the development of future materials that use spatially varying alignment to achieve mechanical functionality beyond conventional layered or unidirectional composites.

## 4 Conclusion

In conclusion, this study uncovers the remarkable complexity and optimization of insect wing design, demonstrating how chitin fiber orientation enhances structural and functional efficiency. Natural patterns of fiber alignment result from millions of years of evolution, balancing crack resistance, flexibility, and energy efficiency in flight. These findings not only deepen our understanding of insect biomechanics but also provide a blueprint for the development of next‐generation micro air vehicles. By integrating material science with evolutionary insights, we can create MAVs that mimic the extraordinary adaptability and resilience of insect wings, advancing the fields of robotics, aerospace engineering, and biomimicry.

## 5 Experimental Section

### Sample Preparation

Locusts *Schistocerca gregaria* were obtained from local pet shops in Kiel, Germany. Hindwings were detached from CO_2_ euthanized adult locusts at least three weeks after the final molt, to ensure proper hardening and sclerotization of the cuticle, and fixed unfolded on a Petri dish (Figure 1a). After air drying for two days, pieces of ≈3 × 3 mm^2^ size were cut from the hindwing using razor blades and mounted ventral side down on 200 µm thick silicon frames of Si_3_N_4_ windows (Norcada Inc., Edmonton, Canada) with a central window sized 2 × 2 mm^2^ as sample holders using two‐component polyvinylsiloxane (President light body, Coltène/Whaledent AG, Altstätten, Switzerland). The 12 regions taken from a single hindwing of one female locust specimen (Figure 1a) were measured in detail using both micro‐ and nano‐focus WAXS. Considering biological variability, two additional locusts (one female and one male) were included, while one region (region 7) was tested for validation. In total, 14 membrane frames were examined.

### Wide‐Angle X‐Ray Scattering (WAXS)

The experiments were performed at the micro‐ and nanoexperimental hutch of the beamline ID13 at the European Synchrotron Research Facility ESRF (Grenoble, France). Samples were mounted on a piezo stage for continuous on‐the‐fly 2D raster scanning. The beam energy was 15.2 keV, corresponding to a wavelength of 0.816 Å. For micro‐focus scanning the X‐ray beam was focused to 2.5 µm. The sample to detector distance was 166 mm. For raster scanning areas of 1.5 × 1.5 mm^2^ the step size was set to 5 µm at an exposure time of 20 ms. The beam size for scanning X‐ray nanodiffraction was ≈200 nm. The detector‐to‐sample distance was 203.5 mm. A lead wire beam stopper and a helium‐filled flight tube were placed between the sample and the detector. The step size of scanning was set to 250 nm with an exposure time of 50 ms per spot. Areas were mapped in square‐shaped patches covering areas of 20 × 20 µm^2^. The X‐ray diffraction signals were recorded using an Eiger X 4 M detector (Dectris Ltd., Baden‐Daetwill, Switzerland). Data were analyzed using customized Python software. For quantitative analyses of both the signal intensity and orientation, the 2D diffraction data were regrouped into the polar coordinate system for determining structural characterizations by featured reflections and their orientations as a function of the scattering vector *q*. In total, data were recorded from 14 pieces of hindwings from three different animals, and among them, sample 9 was used for calibration while samples 1, 3, 5, 7, 10, 11, 12, 13 and 14 were scanned using the nano‐focus setup and samples 2, 4, 6 and 8 were scanned with the micro‐focus setup.

Because of the clearest signal‐to‐noise ratio, the 013 reflection was used for further analysis of the chitin fiber distribution and orientation in this study. The reflection peak of the 013 signal as a function of the scattering vector *q* was reported to be at 18.6 nm^−1^ for α‐chitin in the cuticle of spiders^[^ ^38^ ^]^ and lobster tendon.^[^ ^34^ ^]^ Therefore, for chitin distribution mapping, the intensity of the 013 peak was evaluated in the *q* range between 18 and 19 nm^−1^ in every single diffractogram, each of them representing a single pixel in the signal intensity maps (Figure 2a). The most representative examples were selected for presentation.

To analyze the orientation distribution of chitin fibers, an angular binning approach was applied with 10° intervals. This bin size was chosen to strike a balance between angular resolution and statistical robustness. To further suppress background noise and minimize the influence of weak or disordered signals, a threshold of 500 counts per angular bin was set when identifying dominant orientation peaks (Figure 2). Given that each scanned region contained 6400 individual measurement points, this threshold represents ≈8% of the total dataset per region. Orientations below this threshold were considered statistically insignificant and were excluded from further analysis. This strategy is consistent with methods employed in previous scanning nanofocus X‐ray diffraction studies,^[^ ^36^ ^]^ where intensity‐based filtering was critical for resolving meaningful fiber directions in complex biological materials.

### Finite Element Model Reconstruction and Simulation

A 2D geometrical model (Figure 3a) was reconstructed based on the microscopic images of the locust hind wing and the WAXS results of the membrane. The model was composed of regularly arranged thirty closed cells, which included membrane, longitudinal, and cross veins. Each cell was 1100 µm in length and 600 µm in width, which were the same as the previous measurements.^[^ ^10^ ^]^ Specifically, each cell included half‐wide longitudinal veins (150 µm) at its upper and lower sides, half‐wide cross veins (19 µm) at its right and left sides, and a membrane at its center. The veins were considered hollow circular structures with geometrical characteristics (including diameter and wall thickness) adapted to be equal to the cross‐sectional area and inertia moment of the real morphology. The membrane was further divided into inner, middle, and outer portions based on the imaging results of the WAXS. The three portions of the membrane had the same thickness. Accordingly, four models were reconstructed to represent the natural fiber orientations of the hindwing at the seven different locations (Figure 2). The four models were the same except for the fiber orientations in the inner portion of the membrane. In model I, the fibers in the inner portion were mainly parallel to the longitudinal veins (referring to the membrane at locations 1 and 3). In model II, the fibers in the inner portion were mainly arranged from the anterior‐proximal corner to the posterior‐distal corner of the cell (referring to the membrane at location 7). In model III, the majority of the fibers (approximately 64%) in the inner portion were arranged along the fiber direction of model II, while the rest remained arranged perpendicular to the main direction (referring to the membrane at location 5). In model IV, 76% and 24% of the fibers in the inner portion were arranged parallel and perpendicular to the direction of the longitudinal veins, respectively (referring to the membrane at locations 10, 11, and 12). The fibers in the middle and outer portions of the membrane in models I–IV were arranged at an inclined angle of 45 degrees with respect to the longitudinal veins and perpendicular to the longitudinal veins, respectively. The membrane thicknesses were determined according to the previous measurements,^[^ ^7^ ^]^ where the membranes of models I and II were 4 µm and the membranes of models III and IV were 1.5 µm.

Furthermore, four artificial models were developed to compare the effect of the natural fiber orientations, where the whole membrane was defined as isotropic with random–oriented fibers (model V), anisotropic with fibers along the longitudinal vein (model VI), anisotropic with fibers arranging with an inclined angle of 45 degrees to the longitudinal vein (model VII), and anisotropic with fibers along the cross vein (model VIII), respectively. In order to eliminate the effect of membrane thickness, the membrane thicknesses of the four artificial models were determined to be 4 µm for the comparison with models I and II and 1.5 µm for the comparison with models III and IV. Figure 3b shows the schematic diagram of the fiber orientation in the above eight membrane models.

The mechanical properties of both the longitudinal and cross veins were considered as isotropic linear elastic. The longitudinal veins were assigned an elastic modulus of 3.0 GPa and tensile strength of 52.21 MPa according to the published research.^[^ ^10^, ^16^ ^]^ The elastic modulus and tensile strength of the cross veins were assumed to be 4.0 GPa and 386 MPa based on the previous results of mechanical testing, respectively.^[^ ^50^ ^]^ The fracture toughness and Poisson's ratio of both the longitudinal and cross veins were taken as 1.75 MPa√m and 0.49, respectively.^[^ ^51^ ^]^ Very limited mechanical tests have been performed to clarify the mechanical properties of the membrane, especially considering its anisotropic fibrous microstructures. Based on the current published mechanical data, the elastic moduli *E*_L_ and *E*_T_ were assumed as 1.86 GPa and 369 MPa, representing the mechanical behaviors parallel and perpendicular to the fiber orientation of the membrane, respectively.^[^ ^10^, ^51^ ^]^ The shear modulus was then calculated as $G_{\mathrm{LT}}=\frac{\sqrt{E_{L}E_{T}}}{2(1+\nu_{\mathrm{LT}}\sqrt{\frac{E_{L}}{E_{T}}})}$ based on the Huber's assumption.^[^ ^52^, ^53^ ^]^ *ν*_LT_ is the corresponding Poisson's ratio approximated as 0.49. The longitudinal tensile strength along the fiber orientation *σ*_L_ was taken as the same as that of the longitudinal veins, i.e., 52.21 MPa. Given that the fibrous materials failed under the same strain level regardless of loading direction, the tensile strength perpendicular to the fiber direction *σ*_T_ was calculated to be 10.36 MPa. According to the findings of short‐fiber reinforced polymer composites, the shear strength along the fibers *τ*_LT_ was assumed as half of the transverse tensile strength *σ*_T_, i.e., 5.18 MPa.^[^ ^54^ ^]^

All the above material parameters were used in determining the mechanical properties of the membrane portion with fibers aligned along the longitudinal or cross veins (including the outer portion of models I–IV, the inner portion of model I, and the whole membrane of models VI and VIII). For the membrane portions with fibers mainly aligning neither along the longitudinal nor cross veins (including the middle portions of models I–IV, the inner portions of model II–IV, and the whole membrane of model VII), the elastic moduli and tensile strength of the membrane in global reference axes can be recalculated by using the theory of linear orthotropic elasticity and Tsai‐Hill criterion as^[^ ^54^ ^]^:

(1) $$\begin{array}{ccc} E\left(\theta \right) & & =\left(\frac{\cos^{4}\theta }{\beta E_{L}+\left(1-\beta \right)E_{T}}+\frac{\sin^{4}\theta }{\beta E_{T}+\left(1-\beta \right)E_{L}}+\frac{\sin^{2}2\theta }{4}\right.\\ & & {\left.\left(\frac{1}{{\tilde{G}}_{\mathrm{LT}}}-\frac{2\nu_{\mathrm{LT}}}{\beta E_{L}+\left(1-\beta \right)E_{T}}\right)\right)}^{-1} \end{array}$$

(2) $$\begin{array}{ccc} \sigma \left(\theta \right) & = & \left(\frac{\cos^{4}\theta }{{\left(\beta \sigma_{L}+\left(1-\beta \right)\sigma_{T}\right)}^{2}}+\frac{\sin^{4}\theta }{{\left(\beta \sigma_{T}+\left(1-\beta \right)\sigma_{L}\right)}^{2}}\right.\\ & & {\left.+\frac{\sin^{2}2\theta }{4}\left(\frac{1}{\tau_{\mathrm{LT}}^{2}}-\frac{2\nu_{\mathrm{LT}}}{{\left(\beta \sigma_{L}+\left(1-\beta \right)\sigma_{T}\right)}^{2}}\right)\right)}^{-\frac{1}{2}} \end{array}$$

where *θ* is the angle of fiber orientation in relation to the global axes. *β* is the proportion of the fibers in the main direction with respect to the total fibers. The proportion *β* was set as 1 for the portions with only one fiber direction. Similarly, a modified shear modulus ${\tilde{G}}_{\mathrm{LT}}$ was defined as

(3) $${\tilde{G}}_{\mathrm{LT}}=\frac{\sqrt{\left(\beta E_{L}+\left(1-\beta \right)E_{T}\right)\left(\beta E_{T}+\left(1-\beta \right)E_{L}\right)}}{2\left(1+\nu_{\mathrm{LT}}\sqrt{\frac{\beta E_{L}+\left(1-\beta \right)E_{T}}{\beta E_{T}+\left(1-\beta \right)E_{L}}}\right)}$$

For model V in the fibers were aligned randomly in its membrane, the mechanical properties of the membrane were defined by the Halpin‐Tsai model as

(4) $$E_{R}=\frac{3}{8}E_{L}+\frac{3}{8}E_{T}$$

(5) $$G_{R}=\frac{1}{8}E_{L}+\frac{1}{4}E_{T}$$

(6) $$\nu_{R}=\frac{E_{R}}{2G_{R}}-1$$

(7) σR=2∫0ππ22σθπ

(8) GIC=KIC2KIC2ERER

where *E*_R_, *G*_R_, *ν*_R_, *σ*_R_, and *G*_IC_ are elastic modulus, shear modulus, Poisson's ratio, tensile strength, and work of fracture of the isotropic membrane, respectively. Besides the stiffness and strength of the anisotropic membrane, the work of fracture *G*_IC_ needs to be determined to simulate the anti‐fracture behavior of the hindwing. According to the linear fracture mechanics of anisotropic materials,^[^ ^42^ ^]^ the work of fracture *G*_IC_ for the anisotropic membrane with a longitudinal crack can be calculated as

(9) $$G_{\mathrm{IC}}=K_{\mathrm{IC}}^{2}\sqrt{\frac{1}{2E_{x}E_{y}}}\sqrt{\sqrt{\frac{E_{x}}{E_{y}}}+\frac{E_{x}}{2G_{xy}}-\nu_{xy}}$$

where *E_x_* and *E_y_* are the elastic moduli in the two global reference axes, *G_xy_* is the shear modulus, and *ν*_*xy*_ is Poisson's ratio. The fracture toughness *K*_IC_ of each membrane portion was taken from the published measurements as 0.76 MPa√m, 1.4 MPa√m, and 1.57 MPa√m for the inner, middle, and outer portions, respectively.^[^ ^10^ ^]^ All the material parameters of the eight models are listed in Tables S1 and S2 (Supporting Information). The calculated work of fracture *G*_IC_ along and across the fibers is in coincidence with other previous findings that the fractural work when the crack propagated across the fibers is 8–10 times that when the crack propagated along the fiber direction.^[^ ^51^ ^]^ Based on the above material parameters, the onset of crack in fatigue analyses for the longitudinal veins, cross veins, and different membrane portions has been defined by the following equations:^[^ ^41^]

(10) $$\begin{array}{c} G_{\max}\geq G_{\mathrm{th}}\\ \;\frac{N}{c_{1}{\left(\Delta G\right)}^{c_{2}}}\geq 1 \end{array}$$

where *N* is the number of cycles, Δ*G* is the strain energy release rate (Δ*G* = *G*_max _ − *G*_min _), *G*_th_ and is the threshold value of the strain energy release rate as 1% of the *G*_IC_ values. *c*_1_ and *c*_2_ are empirical material parameters as 5 and −0.0001, respectively. *G*_max _ and *G*_min _ are the maximal and minimal values of the strain energy release rate during the cyclic loading, respectively. The fatigue crack begins to propagate only if both the above equations are satisfied. Subsequently, the rate of crack growth is approximated by the Paris law as:^[^ ^55^ ^]^

(11) $$\frac{da}{dN}=c_{3}{\left(\Delta G\right)}^{c_{4}}\;\;\mathrm{if}\;\;G_{\mathrm{th}}\leq G_{\max}\leq G_{\mathrm{up}}$$

where *a* is the crack growth length, *G*_up_ is the upper limit of the strain energy release rate as 85% of the *G*_IC_ values. *c*_3_ and *c*_4_ are empirical material parameters as $2\times 10^{-17}\frac{m/\mathrm{cycle}}{{\left(m\cdot \mathrm{Pa}\right)}^{6.3}}$ and 6.3, respectively.^[^ ^41^, ^56^ ^]^ When the *G*_max _ reaches the upper limit *G*_up_, a catastrophic crack propagation occurs. Because of a lack of experimental data for the fatigue behaviors of the detailed structures of the wing, all the fatigue coefficients were assumed to be the same for the longitudinal veins, cross veins, and different membrane patterns, except for their critical strain energy release rate *G*_IC_ (listed in Tables S1 and S2, Supporting Information).

The above eight geometrical models (model I–VIII) were meshed by 4‐node bilinear plane stress quadrilateral elements (type CPS4) and simulated using extended finite element methodology (xFEM) in commercial software ABAQUS v6.14/standard (Dassault Systèmes Simulia Corp., Providence, RI, USA). For each model, an initial crack (1.2 mm length) was created at the distal, middle, and proximal ends of the locust hindwings, respectively. In the simulation, the bottom longitudinal vein was fixed in all degrees of freedom, while the upper longitudinal vein was displaced perpendicular to the crack length. In the simulations of fracture under the quasi‐static loads, the hind wing was loaded by an axial tension along its cross veins until failure. Maximal stress theory was chosen for simulating the crack initiation in the FE model of the hindwings, considering the effect of fibers on crack propagation. The curves of displacement reaction force were obtained from each FE simulation and transferred into strain‐stress curves for further comparisons. In the simulations of fatigue under the cyclic loads, the upper longitudinal vein was subjected to cyclic zigzag axial tensile displacement whose maximal and minimal values were 90% and 10% of the ultimate tensile strain (i.e., as 10% based on the simulations and literature results).^[^ ^10^, ^16^ ^]^ A direct cyclic algorithm was used for the fatigue analysis based on the software ABAQUS. The crack length‐cycle number curves for each model were plotted in Figure 3f–h.

In addition, one‐cell FE models were extracted from the above eight models, transferred into the 3D deformable shell, and re‐meshed by 4‐node doubly curved shell elements with reduced integration and hourglass control (type S4R). These one‐cell FE models were used to determine the deformation performance of the different fibrous hindwings under air pressure loads (Figure 5a). The margin of the bottom longitudinal vein was fixed by all degrees of freedom except for its axial rotation. The margin of the upper longitudinal vein was free in both its axial rotation and translation along the direction of the cross vein. Two normal pressure loads (i.e., 12 Pa and 120 Pa) were simulated in the cell model. The lower load (i.e., 12 Pa) corresponds to the high pressure that exists in the locust hindwing during flapping flight.^[^ ^22^ ^]^ Both the deformation and elastic strain energy of the membrane were compared for the eight fibrous patterns. The higher load (i.e., 120 Pa) was used to reveal the potential different failure properties of the different membrane patterns. Considering the anisotropy of the membrane materials, a failure factor was modified from the Tsai–Hill strength theory and used to evaluate the failure differences of the eight fibrous membrane patterns. A failure factor *γ* was defined for the plane stress configuration as

(12) $$\gamma =\sqrt{\frac{\sigma_{1}^{2}}{X^{2}}-\frac{\sigma_{1}\sigma_{2}}{X^{2}}+\frac{\sigma_{2}^{2}}{Y^{2}}+\frac{\tau_{12}^{2}}{S^{2}}}$$

where *σ*_1_ and *σ*_2_ are the tensile stress along the two material reference axes. *τ*_12_ is the shear stress. *X*, *Y* and *S* are the tensile strength in material reference axes and in‐plane shear strength, respectively. The failure factor *γ* increased to 1 when the materials had reached failure.

## Conflict of Interest

The authors declare no conflict of interest.

## Author Contributions

C.L., C.F.S., and S.N.G. conceptualized and designed the research; C.F.S. and S.N.G. supervised the research; C.L., C.F.S., J.L., and M.B. performed the experiments; C.W. performed the simulation; C.L., C.F.S., J.L., and C.W. analyzed the data and wrote the manuscript; C.L., C.F.S., S.N.G., and C.W. discussed the results, reviewed, and edited the manuscript. All authors gave final approval for publication.

## Supporting information



Supporting Information

Supplemental Video 1

Supplemental Video 2

Supplemental Video 3

## Data Availability

The data that support the findings of this study are available from the corresponding author upon reasonable request.
