# Supplementary material for: How Does a Delicate Insect Wing Resist Damage? Chitin Orientation Is Adapted to the Mechanical Demands at the Nanoscale
Source: Adv Mater. 2025 Aug 1;37(51):e03941. doi: 10.1002/adma.202503941 (PMC12721211; doi:10.1002/adma.202503941)
Supplement: Supplementary file 1 — Supporting Information [file ADMA-37-e03941-s002.docx]

Table S1. Material parameters of the four natural membrane models

| Material parameter | Model I | | | Model II | | | Model III | | | Model IV | | |
| --- | --- | --- | --- | --- | --- | --- | --- | --- | --- | --- | --- | --- |
| Portion | Inner | Middle | Outer | Inner | Middle | Outer | Inner | Middle | Outer | Inner | Middle | Outer |
| $E_{x}$/MPa | 1860 | 657 | 369 | 1335 | 657 | 369 | 1323 | 657 | 369 | 1502 | 657 | 369 |
| $E_{y}$/MPa | 369 | 657 | 1860 | 414 | 657 | 1860 | 906 | 657 | 1860 | 727 | 657 | 1860 |
| $E_{z}$/MPa | 369 | 369 | 369 | 369 | 369 | 369 | 369 | 369 | 369 | 369 | 369 | 369 |
| $\nu_{xy}$ | 0.097 | 0.49 | 0.097 | 0.152 | 0.49 | 0.097 | 0.336 | 0.49 | 0.097 | 0.237 | 0.49 | 0.097 |
| $\nu_{xz}$ | 0.097 | 0.275 | 0.49 | 0.135 | 0.275 | 0.49 | 0.137 | 0.275 | 0.49 | 0.120 | 0.275 | 0.49 |
| $\nu_{yz}$ | 0.49 | 0.275 | 0.097 | 0.436 | 0.275 | 0.097 | 0.200 | 0.275 | 0.097 | 0.249 | 0.275 | 0.097 |
| $G_{xy}$/MPa | 340 | 221 | 340 | 292 | 221 | 340 | 389 | 221 | 340 | 390 | 221 | 340 |
| $G_{xz}$/MPa | 340 | 180 | 124 | 279 | 180 | 124 | 278 | 180 | 124 | 299 | 180 | 124 |
| $G_{yz}$/MPa | 124 | 180 | 340 | 134 | 180 | 340 | 220 | 180 | 340 | 192 | 180 | 340 |
| $\sigma_{y}$/MPa | 10.36 | 9.26 | 52.21 | 9.50 | 9.26 | 52.21 | 23.71 | 9.26 | 52.21 | 20.41 | 9.26 | 52.21 |
| $\tau_{xy}$/MPa | 5.18 | 4.63 | 26.11 | 4.75 | 4.63 | 26.11 | 11.85 | 4.63 | 26.11 | 10.20 | 4.63 | 26.11 |
| $G_{\text{IC}}$/$\text{KJ}\cdot\text{m}^{-2}$ | 0.465 | 2.95 | 4.65 | 0.495 | 2.95 | 4.65 | 0.858 | 2.95 | 4.65 | 1.469 | 2.95 | 4.65 |

Note: x, y, and z represent the horizontal, vertical and thickness directions in the global reference axes, respectively.

Table S2. Material parameters of the four membrane models with artificial fiber alignments

| Material parameter | Model V | | | Model VI | | | Model VII | | | Model VIII | | |
| --- | --- | --- | --- | --- | --- | --- | --- | --- | --- | --- | --- | --- |
| Portion | Inner | Middle | Outer | Inner | Middle | Outer | Inner | Middle | Outer | Inner | Middle | Outer |
| $E_{x}$/MPa | 928 | | | 1860 | | | 657 | | | 369 | | |
| $E_{y}$/MPa |  |  |  | 369 | | | 657 | | | 1860 | | |
| $E_{z}$/MPa |  |  |  | 369 | | | 369 | | | 369 | | |
| $\nu_{xy}$ | 0.429 | | | 0.097 | | | 0.49 | | | 0.097 | | |
| $\nu_{xz}$ |  |  |  | 0.097 | | | 0.275 | | | 0.49 | | |
| $\nu_{yz}$ |  |  |  | 0.49 | | | 0.275 | | | 0.097 | | |
| $G_{xy}$/MPa | 325 | | | 340 | | | 221 | | | 340 | | |
| $G_{xz}$/MPa |  |  |  | 340 | | | 180 | | | 124 | | |
| $G_{yz}$/MPa |  |  |  | 124 | | | 180 | | | 340 | | |
| $\sigma_{y}$/MPa | 14.53 | | | 10.36 | | | 9.26 | | | 52.21 | | |
| $\tau_{xy}$/MPa | 7.26 | | | 5.18 | | | 4.63 | | | 26.11 | | |
| $G_{\text{IC}}$/$\text{KJ}\cdot\text{m}^{-2}$ | 2.56 | | | 0.465 | | | 2.95 | | | 4.65 | | |

Note: x, y, and z represent the horizontal, vertical and thickness directions in the global reference axes, respectively.


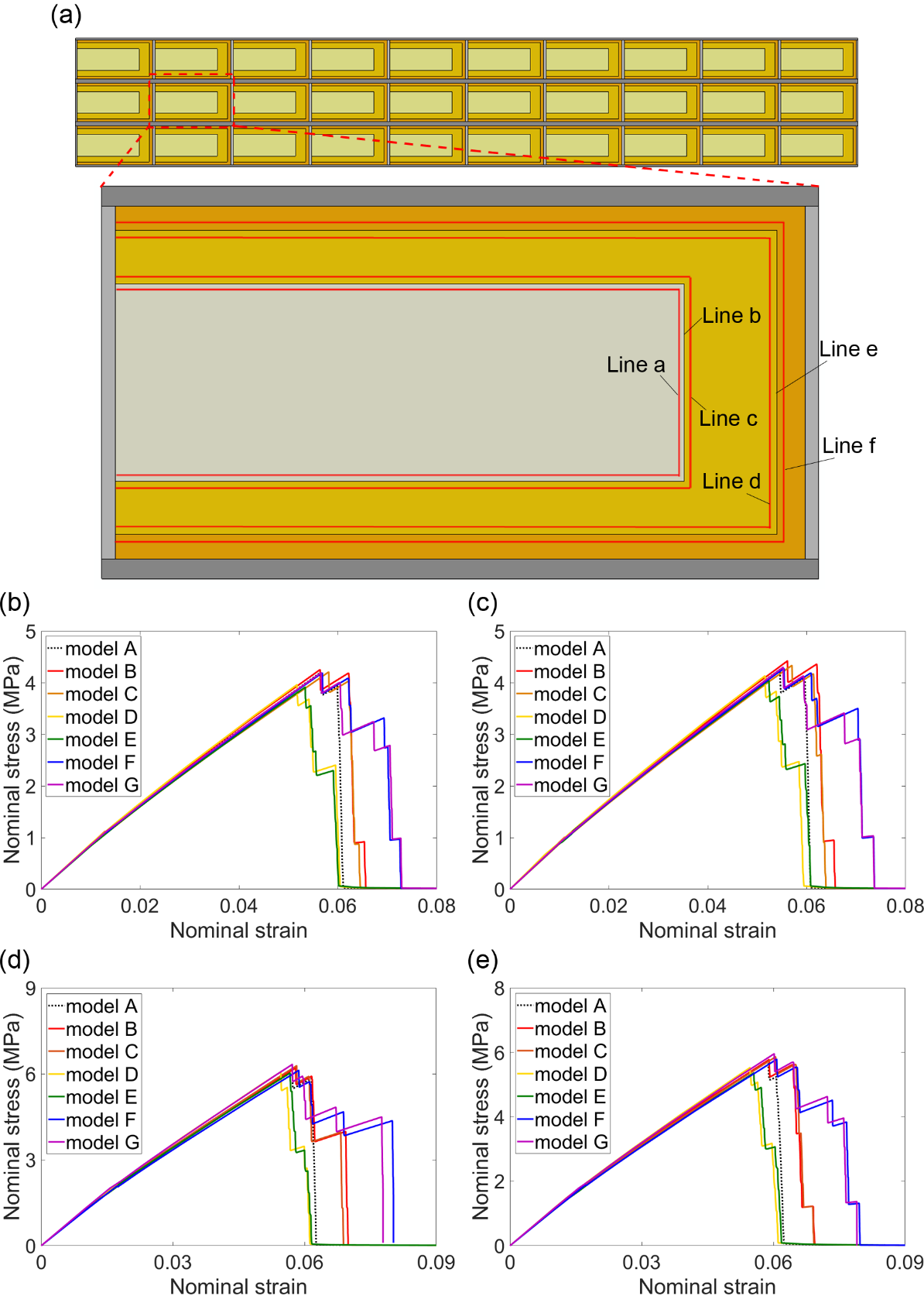


Figure S1. Sensitivity analysis of the geometry parameter of the three membrane portions in the fracture simulations of the four wing models with natural fiber orientations (models I-IV with distal crack). Seven models are constructed with different boundary lines to divide the inner, middle, and outer portions of the membrane: model A (lines b and e), model B (lines a and e), model C (lines c and e), model D (lines b and d), model E (lines b and f), model F (lines a and f), and model G (lines c and d). The distance between the nearby lines is 10 μm. All the aforementioned boundary lines are shown in (a). The comparison between the four original (models I-IV) and its respective modified models with different geometry parameters are shown as follows: (b) model I and its modified models; (c) model II and its modified models; (d) model III and its modified models; (e) model IV and its modified models. The model A in (b-e) is the same as models I-IV (shown in Fig. 3) and is considered the baseline, respectively.


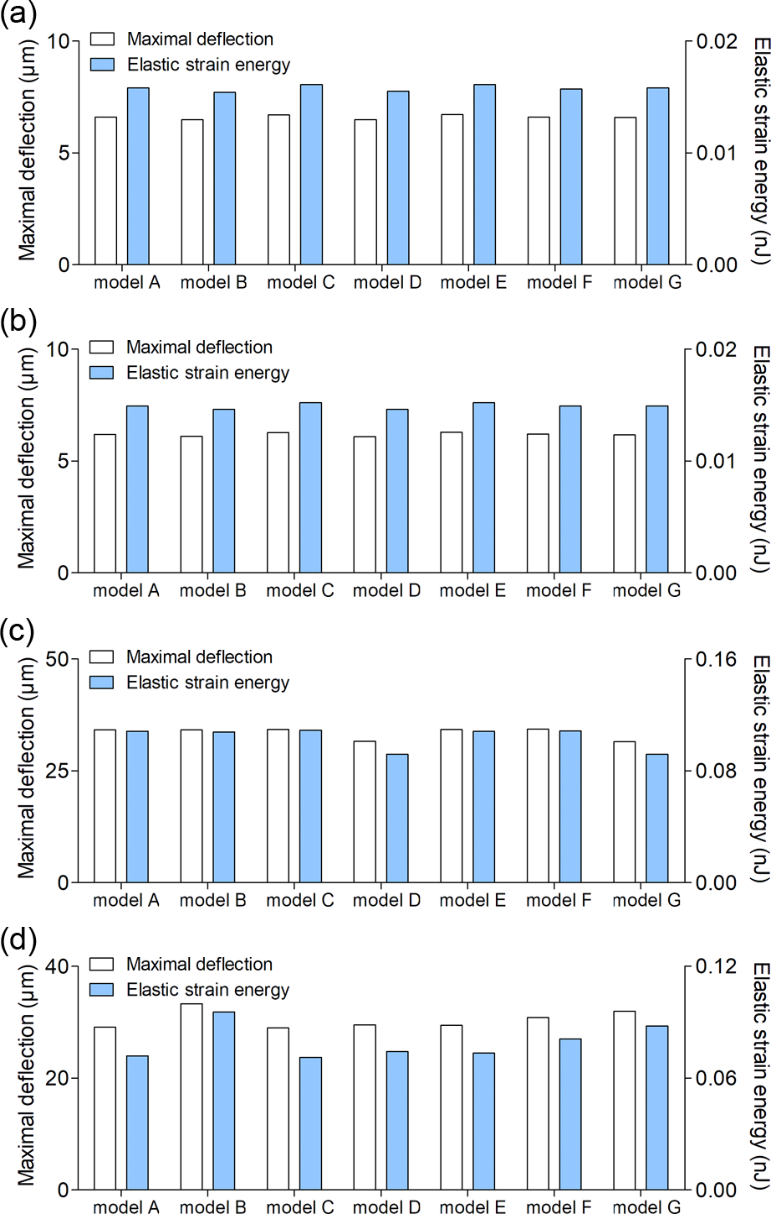


Figure S2. Sensitivity analysis of the geometry parameter of the three membrane portions in the one-cell membrane models under a 12 Pa pressure load. The boundary lines between the three membrane portions in models A-G are the same as those described in Figure S1. The comparisons of maximal deflection and elastic strain energy are shown as follows: (a)model I and its six modified models; (b)model II and its six modified models; (c)model III and its six modified models; (d)model IV and its six modified models. Model A in (a-d) is the same as models I-IV (shown in Fig. 4) and is considered the baseline, respectively.

Video S1. The process of crack propagation for the eight membrane models (I-VIII) under quasi-static tension load when the crack starts at the distal side of the whole model (near the outer margin).

Video S2. The process of crack propagation for the eight membrane models (I-VIII) under quasi-static tension load when the crack starts at the middle site of the whole model.

Video S3. The process of crack propagation for the eight membrane models (I-VIII) under quasi-static tension load when the crack starts at the proximal side of the whole model (near the wing root).
